# Hemoadsorptionin Critically Ill Pediatric Oncology and Hemato-Oncology Patients: A Systematic Review with Structured Narrative Synthesis

**DOI:** 10.3390/children13070961

**Published:** 2026-07-21

**Authors:** Diana Akhmetsharip, Vitaliy Sazonov

**Affiliations:** 1 Department of Medicine, School of Medicine, Nazarbayev University, Astana Z05K4F4, Kazakhstan; 2 Department of Surgery, School of Medicine, Nazarbayev University, Astana Z05K4F4, Kazakhstan; 3 Pediatric Anesthesiology and Intensive Care Unit, National Research Center for Maternal and Child Health, University Medical Center, Astana Z05K4F4, Kazakhstan

**Keywords:** hemoadsorption, hemoperfusion, pediatric oncology, septic shock, cytokine release syndrome, hemophagocytic lymphohistiocytosis, continuous kidney replacement therapy

## Abstract

**Highlights:**

**What are the main findings?**

• Across 12 published reports, hemoadsorption was most often described as adjunctive or rescue therapy for septic shock, sepsis-like hyperinflammation, secondary hemophagocytic lymphohistiocytosis, CAR-T-cell-associated cytokine release syndrome, delayed methotrexate clearance, and transplant-related complications in critically ill pediatric oncology or hemato-oncology patients.
• Direct pediatric oncology evidence was limited to uncontrolled case reports, case series, and small observational cohorts; these reports described short-term before–after reductions in inflammatory biomarkers and improvements in physiologic variables, but these changes could not be separated from concurrent intensive care interventions.

**What are the implications of the main findings?**

• Hemoadsorption should be interpreted as an individualized adjunctive rescue intervention that has been attempted in selected critically ill children with cancer, not as a standard therapy with proven survival benefit.
• Future studies should use syndrome-specific eligibility criteria, standardized outcome timing, independent safety reporting, patient-overlap checks, anticoagulation reporting, and therapeutic drug monitoring because nonselective drug adsorption remains a major unresolved safety concern.

**Abstract:**

**Background**: Critically ill children with cancer are vulnerable to sepsis and septic shock, secondary hemophagocytic lymphohistiocytosis (HLH), cytokine release syndrome, delayed chemotherapy clearance, and multiorgan dysfunction. Although hemoadsorption can remove inflammatory mediators and selected toxins, evidence in pediatric oncology remains limited and heterogeneous. **Objective**: To systematically review the indications, technical application, biomarker and physiologic findings, safety reporting, and clinical outcomes of hemoadsorption in critically ill pediatric oncology and hemato-oncology patients. **Methods**: We conducted a PRISMA-guided systematic review of PubMed, Scopus, and Web of Science from January 2017 to 1 June 2026, supplemented by reference screening. The search strategy was expanded to capture cartridge-based hemoadsorption and related extracorporeal blood purification modalities, including albumin dialysis systems, but direct synthesis was restricted to cartridge-based hemoadsorption. Evidence was stratified as direct pediatric oncology evidence, supportive hemato-oncology/transplant evidence, or contextual mixed pediatric critical-care evidence. Because included reports were small, uncontrolled, and clinically heterogeneous, synthesis followed a structured narrative approach without meta-analysis. **Results**: Twelve reports met the inclusion criteria; most were case reports, case series or retrospective observational studies. Hemoadsorption was most often described for septic shock, sepsis-like hyperinflammation, or secondary HLH, with smaller experience in delayed methotrexate clearance, CAR-T-cell-associated cytokine release syndrome, and post-transplant hyperbilirubinemia. Reported devices included CytoSorb, Jafron HA330, and HA230. Direct pediatric oncology reports described before–after reductions in IL-6, IL-10, C-reactive protein, procalcitonin, or ferritin, together with changes in oxygenation, vasoactive support, or organ dysfunction scores. However, all findings were vulnerable to confounding by concurrent antimicrobials, immunomodulation, kidney replacement therapy, source control, and natural recovery. Mortality outcomes were reported using non-equivalent horizons and were summarized narratively. **Conclusions**: Hemoadsorption has been attempted as adjunctive rescue support in selected critically ill pediatric oncology and hemato-oncology patients. Current evidence is insufficient to determine treatment effect, survival benefit, optimal timing, device selection, anticoagulation strategy, or pharmacokinetic safety.

## 1. Introduction

Advances in diagnosis, chemotherapy, immunotherapy, transplantation, and supportive care have substantially improved survival in childhood cancer [1]. As survival improves, pediatric intensive care units (PICUs) are managing more children with cancer who develop complex critical illness rather than isolated organ failure [2,3]. This population is at increased risk for sepsis, refractory shock, cytokine release syndrome (CRS), hemophagocytic lymphohistiocytosis (HLH), acute kidney injury, liver dysfunction, and multiorgan dysfunction due to immune suppression, neutropenia, mucosal barrier injury, tumor biology, treatment-related toxicity, and transplantation-related complications [3,4,5]. Recent pediatric oncology sepsis data suggest that these children require more sustained vasoactive support and experience more new morbidity at discharge than septic children from other specialties, even when mortality is comparable.

A shared pathophysiological feature across many of these syndromes is dysregulated systemic inflammation. Excessive circulating cytokines, including interleukin (IL)-6, IL-8, IL-10, tumor necrosis factor-alpha, interferon-gamma, and other damage-associated molecular patterns, can promote endothelial dysfunction, vasoplegia, capillary leak, mitochondrial dysfunction, coagulopathy, and progressive organ failure [6,7,8]. In pediatric oncology, this inflammatory burden may be amplified by pre-existing renal, hepatic, pulmonary, or cardiac vulnerability from malignancy or its treatment. Conventional management remains centered on antimicrobials, source control, immunomodulation, hemodynamic support, mechanical ventilation, kidney replacement therapy, and extracorporeal membrane oxygenation (ECMO) when indicated [9]. While these strategies are life-saving, they do not always rapidly reduce the broad mediator burden seen in refractory hyperinflammatory states [8,10].

Hemoadsorption is an extracorporeal blood purification technique in which whole blood or plasma is passed through a cartridge containing a sorbent material that can bind middle-molecular-weight inflammatory mediators and certain toxins. Cartridge-based systems, such as the CytoSorb and Jafron HA-series devices, can be used as standalone hemoadsorption devices or integrated into continuous kidney replacement therapy (CKRT), continuous veno-venous hemodiafiltration (CVVHDF), or extracorporeal membrane oxygenation (ECMO) circuits [10,11,12,13]. This approach is particularly attractive in pediatric onco-critical care because nonselective, broad-spectrum mediator removal could complement supportive care when inflammation is severe, rapidly progressive, or refractory to targeted pharmacologic therapy. However, this same nonselectivity could also remove essential antimicrobials, antifungals, immunosuppressive drugs, and chemotherapeutic agents, which is a particular concern for children receiving multidrug oncology regimens [13,14].

Although hemoadsorption is increasingly discussed in pediatric critical care, evidence specific to pediatric oncology and hemato-oncology remains scattered across case reports, small case series, mixed pediatric intensive care unit (PICU) cohorts, and transplant-related publications. The degree to which reported biomarker reductions reflect hemoadsorption rather than concurrent intensive care interventions is uncertain. The objective of this systematic review was therefore to synthesize published clinical evidence on cartridge-based hemoadsorption in critically ill pediatric oncology and hemato-oncology patients, while clearly distinguishing direct pediatric oncology evidence from supportive transplant-associated and contextual mixed pediatric critical-care evidence. The review question was: in children with malignancy, hemato-oncology conditions, transplant-associated complications, or immunotherapy-related toxicity who receive cartridge-based hemoadsorption during critical illness, what indications, technical approaches, biomarker or physiologic changes, safety concerns, and clinical outcomes have been reported?

## 2. Materials and Methods

### 2.1. Protocol and Reporting

This review was conducted and reported in accordance with the Preferred Reporting Items for Systematic Reviews and Meta-Analyses (PRISMA) 2020 statement and the PRISMA-S extension for search reporting [15,16]. Narrative synthesis was structured using Synthesis Without Meta-analysis (SWiM) principles because meta-analysis of treatment effects was not appropriate given the small, uncontrolled, clinically heterogeneous evidence base [17]. The review was registered in PROSPERO (CRD420261412203) on 1 June 2026. The protocol can be accessed through the PROSPERO registry using this registration number.

During revision, three clarifications were made in response to peer review. First, the search strategy was expanded to include albumin dialysis and extracorporeal liver support terms, including MARS, Prometheus, SPAD, and albumin dialysis, to document whether relevant liver-support literature had been captured. Second, evidence categories were operationalized as direct, supportive, or contextual according to population and extractability of pediatric oncology-specific outcomes. Third, the initially planned exploratory pooling of mortality proportions was removed because outcome horizons were inconsistent, included reports were uncontrolled and highly selected, and possible overlap between reports from the same center could not be excluded with sufficient certainty. These changes did not alter the central review question but changed the presentation of the synthesis from exploratory quantitative synthesis to structured narrative synthesis.

### 2.2. Search Strategy and Information Sources

From 1 January 2017 through 1 June 2026, we searched PubMed, Scopus, and Web of Science. The start date of 2017 was chosen to include contemporary pediatric reports that use current hemoperfusion technologies, while limiting the influence of older devices and earlier supportive care practices. The search was restricted to English-language reports due to the unavailability of translation resources. This restriction is acknowledged as a potential source of language bias. Reference lists of eligible articles and relevant reviews were also screened.

The search combined three concepts: (1) hemoadsorption, hemoperfusion, and related extracorporeal blood purification technologies; (2) pediatric populations; and (3) oncology, hemato-oncology, transplant, immunotherapy, sepsis, hyperinflammation, chemotherapy toxicity, or liver dysfunction contexts. To address extracorporeal liver support, the search was broadened to include albumin dialysis, MARS, Prometheus, fractionated plasma separation and adsorption, SPAD, and single-pass albumin dialysis. These liver-support modalities were not considered direct cartridge-based hemoadsorption evidence unless the report included cartridge-based hemoadsorption, but they were screened to identify relevant contextual pediatric oncology or transplant-associated detoxification reports. Complete line-by-line strategies for each database, including field tags and limits, are provided in Supplementary Materials (Supplementary S1).

### 2.3. Eligibility Criteria

The eligibility criteria were defined using a PICOS framework. The population of interest included patients aged 0–18 years with malignant or oncohematologic diseases, those undergoing hematopoietic stem cell transplantation or CAR-T cell therapy, and those with clinically relevant, transplant- or immunotherapy-associated critical illness. Mixed pediatric cohorts were only eligible if oncology-relevant data could be extracted separately or if the report provided contextual information on hemoadsorption feasibility, safety, extracorporeal circuit integration, or related extracorporeal blood purification approaches.

The intervention of interest was cartridge-based hemoadsorption or hemoperfusion, including CytoSorb and Jafron HA-series cartridges. It could be delivered as a standalone intervention or integrated into continuous renal replacement therapy, continuous kidney replacement therapy, continuous venovenous hemodiafiltration, extracorporeal membrane oxygenation, or another extracorporeal circuit. A comparator was not required because the anticipated evidence base consisted largely of case reports, case series, and uncontrolled observational studies. However, comparative studies were included when available.

Eligible outcomes included reported changes in inflammatory or disease-specific biomarkers, physiologic variables, organ support variables, and survival or mortality with a clearly reported timeframe. Other eligible outcomes included renal or hepatic recovery, treatment feasibility, anticoagulation, circuit complications, bleeding, thrombocytopenia, drug concentrations, and other adverse events. Eligible study designs included case reports, case series, retrospective and prospective observational studies, nonrandomized interventional studies, and mixed pediatric cohorts with relevant, extractable data. We excluded reviews, editorials, mechanistic reports without pediatric clinical data, adult-only reports, conference abstracts without sufficient patient-level information, and reports without extractable clinical outcomes.

Reports were stratified into direct, supportive, and contextual evidence before synthesis. Direct evidence referred to reports with extractable pediatric oncology or malignant hematology patients who received cartridge-based hemoadsorption. Supportive evidence referred to hematology, transplant-associated, immunotherapy-associated, or partially oncologic pediatric reports in which the findings were clinically relevant, yet not entirely separable for malignant disease. Contextual evidence referred to mixed pediatric critical care or extracorporeal blood purification reports that informed the feasibility, safety, device integration, or alternative extracorporeal approaches, but that were not used as direct pediatric oncology evidence.

We excluded albumin dialysis systems (including MARS, Prometheus, and single-pass albumin dialysis), other liver-support approaches, therapeutic plasma exchange, high-flux hemodialysis, continuous renal replacement therapy without a hemoadsorption cartridge, and adsorptive membranes (such as oXiris) from the direct hemoadsorption synthesis unless cartridge-based hemoadsorption was also used. These modalities were included only for contextual discussion when relevant to pediatric oncology, transplant-associated toxicity, or extracorporeal detoxification.

### 2.4. Study Selection, Data Extraction, and Outcomes

Titles and abstracts were screened against prespecified eligibility criteria, followed by full-text review. Two reviewers independently screened titles, abstracts, and full texts, extracted data using a standardized form. The form was tested on two eligible reports and refined before completion of extraction. Extracted variables included study year, country, center, recruitment period, design, patient age, sex and weight where available, oncologic or hemato-oncologic diagnosis, treatment status, indication for hemoadsorption, severity markers, device, cartridge number, circuit configuration, priming approach, timing and duration of therapy, anticoagulation, concurrent therapies, biomarkers, organ support variables, adverse events, follow-up duration, funding, conflicts of interest, and clinical outcomes.

Because several reports had overlapping authors, centers, or clinical settings, we assessed possible patient overlap by comparing author groups, institutions, recruitment periods, ages, diagnoses, indications, dates, and outcome descriptions. When overlap could not be ruled out, the reports were linked narratively and not combined into a pooled denominator. This approach was used to reduce the risk of double-counting patients across overlapping reports, an important source of bias in evidence synthesis when multiple publications may include the same or partially overlapping populations [18]. We did not contact study authors for additional unpublished patient-level data, which is acknowledged as a limitation.

Outcomes were extracted separately rather than combined into a composite. Biomarker outcomes included reported changes in IL-6, IL-10, C-reactive protein (CRP), procalcitonin (PCT), ferritin, bilirubin, methotrexate concentration, and other disease-specific markers. Physiologic outcomes included the vasoactive-inotropic score, vasoactive dose, oxygenation indices, lactate levels, urine output, kidney and liver function, and organ dysfunction scores. Safety outcomes included bleeding, thrombocytopenia, transfusion, hemodynamic intolerance, circuit clotting, catheter complications, treatment interruption, cartridge failure, and changes in drug concentrations. It was also noted whether adverse events were actively assessed. Mortality and survival outcomes were extracted exactly as reported. These included PICU mortality, hospital mortality, 28-day mortality, survival to ward transfer, survival to discharge, and later death from malignancy progression. These outcome horizons were not pooled.

### 2.5. Risk of Bias and Certainty Assessment

Risk of bias was assessed according to study design. Case reports and case series were appraised using Joanna Briggs Institute critical appraisal checklists for case reports and case series [19]. Nonrandomized comparative or interventional studies were interpreted using ROBINS-I domains, and retrospective cohort studies were evaluated using Newcastle-Ottawa Scale concepts [20,21]. Given the predominance of small uncontrolled designs, heterogeneity of indications, and absence of robust comparators, certainty was judged qualitatively rather than by formal GRADE rating. Because one of the authors of this review was also an author of several of the included reports, a reviewer who was not involved in the primary studies appraised those reports and then checked for consistency. Domain-level judgments and supporting comments are provided in Supplementary Table S1. We did not convert checklist responses into global quality labels when the tool did not provide an overall rating. We did not formally grade certainty using GRADE because the included evidence consisted predominantly of uncontrolled case reports, case series, and small retrospective cohorts with heterogeneous indications and outcomes. Instead, we described confidence in the evidence qualitatively by outcome domain, considering study design, directness, consistency, risk of bias, reporting completeness, possible publication bias, and confounding by co-interventions.

### 2.6. Data Synthesis and Statistical Analysis

Because studies differed substantially in population, indication, device, circuit configuration, timing, co-interventions, and outcome definitions, the primary synthesis was structured narrative synthesis rather than meta-analysis. The narrative synthesis followed SWiM principles [17]. First, reports were grouped by evidence category (direct, supportive, or contextual). Then, they were grouped by clinical syndrome or therapeutic target. The syndromes and targets were: sepsis/septic shock and sepsis-like hyperinflammation; HLH-like hyperinflammation; delayed methotrexate clearance or chemotherapy-related toxicity; CAR-T-cell-associated cytokine release syndrome or immune-effector toxicity; and transplant-related hyperbilirubinemia or liver dysfunction. Within each group, we summarized the following: study design; population; device; timing; co-interventions; biomarker findings; physiologic findings; safety reporting; and outcome horizon.

We described the direction of reported biomarker or physiologic change only as a before–after observation within uncontrolled reports. We did not infer a treatment effect from temporal changes, nor did we use vote-counting to determine efficacy. Conflicting findings, incomplete reporting, and major co-interventions were described narratively. Mortality and survival outcomes were summarized by report and by reported outcome horizon. We did not calculate a pooled mortality estimate because the reports used non-equivalent outcome horizons, included highly selected rescue cases, lacked comparable untreated groups, and had potential patient overlap across centers. No imputation was performed for missing data.

## 3. Results

### 3.1. Study Selection and Characteristics

**Figure 1:**
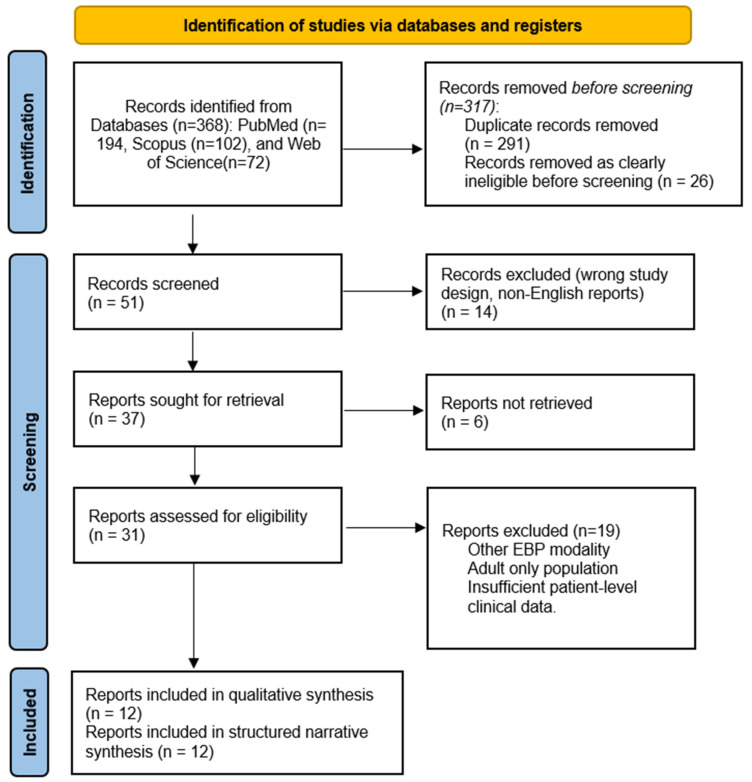
PRISMA 2020 flow diagram for study selection. (The flow diagram summarizes database searching, screening, full-text eligibility assessment, and final inclusion of studies in the qualitative synthesis).

The initial search identified 368 records. After duplicate removal and removal of clearly ineligible records before screening, 51 records underwent title and abstract screening. Fourteen records were excluded at title and abstract screening. Thirty-seven reports were sought for retrieval, of which six could not be retrieved. Thirty-one reports underwent full-text assessment. Nineteen full-text reports were excluded for the following reasons: adult-only population, extracorporeal blood purification without cartridge-based hemoadsorption, review or background article without extractable clinical data, insufficient patient-level or subgroup data, or non-oncology/non-contextual populations. Twelve reports were included in the qualitative synthesis. The PRISMA 2020 flow diagram is shown in Figure 1.

The included evidence consisted mainly of case reports, case series, and retrospective observational studies (Table 1). Direct pediatric oncology evidence was concentrated in sepsis, refractory septic shock, sepsis-triggered HLH, neutropenic shock, delayed methotrexate clearance, and CAR-T-cell-associated CRS [22,23,24,25,26,27,28,29,30,31]. Supportive evidence came from pediatric hyperbilirubinemia, mixed critically ill pediatric cohorts, and studies that included hemato-oncology or transplant subgroups [28,29,32,33]. Across included reports, the most frequent malignancies or oncologic contexts were acute lymphoblastic leukemia, acute myeloid leukemia, lymphoma, hematopoietic stem cell transplantation, and CAR-T therapy.

Of the 12 included reports, 7 provided direct, separately extractable pediatric oncology or malignant hemato-oncology evidence [22,23,24,25,26,30,31]; 4 were supportive hemato-oncology, transplant-associated, or partially oncologic reports [27,28,29,32]; and 1 was contextual mixed pediatric critical-care evidence [33]. The 7 direct reports described 27 report-level pediatric oncology or malignant hemato-oncology cases/patients before accounting for possible overlap. This number should not be interpreted as a confirmed unique-patient denominator because possible overlap between reports from the same Astana pediatric intensive-care setting could not be excluded with certainty. Mixed-cohort findings were not interpreted as direct pediatric oncology outcomes unless oncology-specific data were separately extractable. Where overlap could not be excluded, reports were linked narratively and were not combined into a pooled denominator.

### 3.2. Indications and Technical Application

Three clinical indication clusters were identified. First, hemoadsorption was used for septic shock, sepsis-like cytokine storm, MODS, and secondary HLH in immunocompromised oncology patients [22,23,24,25,26,27]. This was the largest and most clinically coherent direct oncology subgroup. Second, hemoadsorption was applied for treatment-related toxicities, including delayed high-dose methotrexate clearance and CAR-T-cell-associated CRS or acute respiratory distress syndrome [30,31]. Third, hemoadsorption was used in transplant-related hyperbilirubinemia or mixed pediatric critical illness, providing contextual safety and feasibility information but less direct oncology-specific inference [32,33].

CytoSorb and Jafron HA330 were the most frequently reported devices. CytoSorb was commonly integrated into CRRT, CKRT, CVVHDF, or ECMO-adjacent extracorporeal support; HA330 was reported largely in septic and neutropenic shock. HA230 was used in delayed methotrexate clearance after high-dose chemotherapy [30]. Heparin was the most common anticoagulant, although regional citrate was reported or proposed in situations with high bleeding risk or thrombocytopenia [24,34]. Treatment duration ranged from single extended sessions to multi-day protocols with cartridge exchange, typically every 24 h.

### 3.3. Biomarker and Physiologic Response

Across direct pediatric oncology reports, authors described before–after reductions in inflammatory biomarkers after hemoadsorption, including IL-6, IL-10, CRP, PCT, and ferritin, together with reported changes in oxygenation, vasoactive support, or organ dysfunction scores (Table 2). These findings should be interpreted as temporal observations rather than treatment effects. In most reports, hemoadsorption was delivered alongside multiple co-interventions, including antimicrobials, source control, immunomodulation, CRRT or CKRT, fluid management, transfusion support, and organ support. Therefore, the independent contribution of hemoadsorption could not be isolated. In the largest direct oncology cohort, Ryazanova et al. compared CytoSorb and HA330 in 20 pediatric oncology patients with sepsis and reported marked decreases in CRP, procalcitonin (PCT), and IL-6 in both groups, along with improved SpO_2_/FiO_2_ ratio, lower norepinephrine dose, and improved pediatric Sequential Organ Failure Assessment score [22]. No statistically significant between-device superiority was demonstrated.

Sazonov et al. reported HA330 hemoadsorption in septic pediatric cancer patients with rapid post-session reductions in CRP, PCT, and IL-6, improvement in oxygenation, reduced vasoactive support, and recovery of urine output in responding cases [23]. In a child with acute myeloid leukemia, Fanconi anemia, invasive fungal disease, severe thrombocytopenia, and hyper-IL-6-naemia, CytoSorb integrated with CRRT and citrate anticoagulation was associated with biomarker reduction and clinical stabilization despite substantial hematologic vulnerability [24]. In pediatric HLH, CytoSorb hemoperfusion was associated with reductions in IL-10, PCT, and ferritin, although improvements in vasoactive-inotropic score and PELOD were not statistically significant in the small cohort [25].

### 3.4. Reported Survival and Mortality Outcomes

The included literature reported survival and mortality outcomes inconsistently and did not pool them. The outcome horizons included survival to ward transfer, PICU or hospital discharge, 28-day or hospital mortality, and later death related to progression of the underlying malignancy. These endpoints are clinically distinct and cannot be interpreted as a single acute mortality outcome. Additionally, possible overlap between reports from the same institution or author group could not be sufficiently excluded for a pooled, denominator-based analysis. Therefore, survival and mortality are summarized descriptively by report in Table 3.

In the largest direct pediatric oncology cohort, Ryazanova et al. reported outcomes in a mixed oncology population treated for sepsis or multiple organ dysfunction syndrome (MODS); deaths were described in relation to the longer-term oncologic course rather than as a uniform acute hemoadsorption endpoint [22]. Sazonov et al. reported two leukemia cases in the oncology-relevant subset, including one later death from refractory malignancy [23]. Single-patient reports described survival to ward transfer or discharge; however, these selected rescue cases cannot be used to estimate treatment effects or prognosis. Overall, the available data do not establish whether hemoadsorption improves survival.

### 3.5. Safety, Anticoagulation, and Drug Adsorption

Safety reporting was incomplete and did not allow reliable estimation of adverse-event rates. Several reports stated that hemoadsorption was technically feasible, including in children with thrombocytopenia or complex extracorporeal support, but most case reports and case series did not systematically define or actively ascertain adverse events. Reported or extractable safety domains included anticoagulation approach, bleeding, thrombocytopenia, circuit clotting, treatment interruption, hemodynamic intolerance, catheter complications, transfusion needs, and drug concentration monitoring, but these were inconsistently reported across studies. Therefore, absence of reported device-related serious adverse events should not be interpreted as evidence of safety [24,34].

A central oncology-specific concern is nonselective drug removal. Hemoadsorption cartridges may adsorb antibacterial, antifungal, immunosuppressive, immunomodulatory, or chemotherapeutic agents, potentially causing underexposure during life-threatening infection or narrow therapeutic-margin oncology treatment [35,36]. The magnitude of this risk in children with cancer is poorly quantified. When hemoadsorption is used in pediatric oncology, therapeutic drug monitoring, dose reassessment, and close coordination among intensivists, oncologists, nephrologists, infectious disease specialists, and pharmacists should be considered whenever feasible.

## 4. Discussion

This systematic review summarizes a small and heterogeneous body of evidence describing hemoadsorption in critically ill pediatric oncology and hemato-oncology patients. The available reports show that hemoadsorption has been attempted most often as adjunctive rescue therapy in severe sepsis, sepsis-like hyperinflammation, HLH-like syndromes, CAR-T-cell toxicity, delayed methotrexate clearance, and transplant-related complications. The most consistent observations were before–after reductions in inflammatory biomarkers and selected physiologic variables [22,23,24,25,26,27,28,29]. However, these observations arose largely from uncontrolled reports with major concurrent interventions and should not be interpreted as proof of treatment efficacy. The current evidence cannot determine whether hemoadsorption independently improves survival, prevents organ failure, reduces organ-support duration, or improves long-term outcomes.

A central interpretive issue is the gap between biomarker response and patient-centered outcomes. The observed reductions in cytokines and acute-phase markers are mechanistically consistent with the adsorptive properties of CytoSorb, HA-series cartridges, and related extracorporeal blood purification platforms [7,8,10,11,12,13]. However, biomarker improvement does not necessarily equal clinical recovery [37]. Cytokine concentrations are dynamic, compartmentalized, and influenced by source control, antimicrobial treatment, immunomodulation, renal replacement therapy, fluid balance, and spontaneous disease evolution [38]. In addition, patients who survive long enough to complete hemoadsorption may be systematically different from those with rapidly irreversible shock, introducing survivor and indication bias [39]. This concern is consistent with broader critical care literature cautioning against routine extracorporeal hemoadsorption outside carefully selected rescue contexts [14]. For this reason, the most defensible conclusion is that published reports describe temporal biomarker and physiologic changes after hemoadsorption, while the independent contribution of hemoadsorption to clinical recovery or mortality remains unknown.

The pediatric oncology context makes this distinction especially important. Children with malignancies differ from general PICU populations because of chemotherapy-related marrow suppression, mucosal barrier injury, neutropenia, prior organ toxicity, invasive devices, exposure to broad-spectrum anti-infective therapy, and, increasingly, immunotherapy-associated hyperinflammation [1,2,3,4]. These factors increase susceptibility to infection, shock, bleeding, and treatment-related toxicity, while also making clinical attribution difficult [40]. In the included reports, hemoadsorption was usually applied as rescue or adjunctive therapy in patients who were already critically ill, rather than as an early standardized intervention. Thus, apparent improvement after hemoadsorption cannot be separated from concurrent intensive care measures in most studies. At the same time, the repeated feasibility signal across different centers and devices is meaningful for pediatric critical care, because extracorporeal therapies in small children require attention to vascular access, circuit volume, hemodynamic tolerance, cartridge exchange, priming, and anticoagulation [10,11,12,26,33].

Timing remains a clinically important but unresolved question. While several reports describe hemoadsorption initiation within the first 12–24 h of refractory shock or hyperinflammatory deterioration, others describe its use as a later rescue therapy after the development of multiorgan dysfunction. [22,25,29]. A biologically plausible hypothesis is that mediator removal might be more relevant before irreversible endothelial injury, mitochondrial dysfunction, microcirculatory failure, and multiorgan injury become fixed [41]. However, the available reports do not allow for a valid comparative analysis of early versus late initiation. Apparent associations between earlier use and favorable outcomes are vulnerable to confounding factors such as disease severity, center expertise, patient selection, and survivor bias. Therefore, timing should be treated as a hypothesis for future study rather than a conclusion of this review. Future studies should prespecify objective initiation triggers, such as the vasoactive-inotropic score, lactate trajectory, the oxygenation index, the pSOFA or the PELOD-2 progression, ferritin or IL-6 thresholds, refractory capillary leak, or failure of conventional sepsis- or HLH-directed therapy.

Device choice and circuit configuration remain insufficiently studied. CytoSorb was the most frequently reported device across pediatric sepsis, HLH, CAR-T toxicity, and mixed critical-care contexts; HA330 was reported mainly in pediatric oncology septic shock; and HA230 was reported for delayed methotrexate clearance [22,23,25,28,29,30,31,32]. The direct pediatric oncology comparison by Ryazanova et al. found that both HA330 and CytoSorb were followed by biomarker and physiologic changes, but the study was small, nonrandomized, and did not establish device superiority [22]. Therefore, device selection should be described as a technical and pharmacologic decision under uncertainty rather than as an evidence-based recommendation. Relevant considerations include the intended therapeutic target, patient size, vascular access, cartridge availability, circuit compatibility, institutional experience, anticipated adsorption spectrum, and ability to monitor drug concentrations [22,23,25,30,31].

Anticoagulation is another practical issue that deserves explicit discussion in pediatric oncology. Heparin was commonly reported in the included studies, but systemic anticoagulation may be problematic in children with thrombocytopenia, coagulopathy, recent procedures, invasive fungal disease, or bleeding risk [42,43]. Regional citrate anticoagulation may reduce systemic anticoagulant exposure during CKRT and has been described as a feasible alternative in critically ill children, although it requires careful monitoring of ionized calcium, acid-base status, liver function, and circuit performance [24,29,34]. The current review cannot determine whether citrate, heparin, or no anticoagulation is preferable for hemoadsorption in pediatric oncology, because anticoagulation strategies were not compared systematically. Future reports should standardize reporting of anticoagulation method, platelet trends, bleeding events, circuit clotting, cartridge downtime, transfusion requirements, and reasons for treatment discontinuation.

Safety assessment must extend beyond procedural complications. A major oncology-specific concern is non-selective adsorption of concomitant drugs. Hemoadsorption can potentially remove not only inflammatory mediators but also anti-infective agents, antifungals, immunosuppressants, immunomodulators, and selected chemotherapy-related compounds [35,36]. This risk is particularly relevant in pediatric oncology because underexposure to antimicrobials during neutropenic sepsis or invasive fungal disease may be clinically consequential, while unintended removal of immunomodulatory or chemotherapeutic agents could alter treatment efficacy or toxicity [44,45]. Conversely, in delayed methotrexate clearance, drug removal is the intended therapeutic target [30,46]. These opposing pharmacokinetic implications highlight the need to analyze indication-specific safety. Whenever feasible, hemoadsorption protocols in pediatric oncology should include therapeutic drug monitoring, dose reassessment after cartridge initiation or exchange, documentation of drug timing relative to hemoadsorption sessions, and multidisciplinary input from intensivists, oncologists, nephrologists, pharmacists, and infectious disease specialists.

The potential role of hemoadsorption differs substantially by syndrome. In septic shock and HLH-like hyperinflammation, the therapeutic goal is reduction in mediator burden, vasoplegia, capillary leak, and organ dysfunction [22,23,24,25,26,27,28,29]. In delayed methotrexate clearance, the objective is accelerated toxin removal and mitigation of renal or hepatic injury [30,46]. In CAR-T-cell-associated cytokine release syndrome or immune-effector toxicity, hemoadsorption should be viewed as a rescue adjunct to established immunomodulatory strategies rather than a replacement for tocilizumab, corticosteroids, anakinra, CKRT, therapeutic plasma exchange, or adsorptive membranes such as oXiris [31,47,48,49]. In hyperbilirubinemia after transplantation or severe liver dysfunction, the target is bilirubin removal and possibly extracorporeal liver support, but the evidence remains indirect for pediatric oncology and should not be merged with sepsis outcomes [32]. Combining these indications into a single efficacy claim risks overstating the evidence; future studies should stratify analyses by clinical syndrome and therapeutic target.

Comparison with other extracorporeal blood purification approaches is also necessary for clinical positioning. High-flux hemodialysis and CKRT are established for small-solute clearance, fluid control, and support of renal dysfunction, and may be useful in delayed methotrexate excretion [46]. Therapeutic plasma exchange may remove circulating inflammatory mediators and immune complexes but requires plasma replacement and carries different bleeding, allergic, and volume-related risks [50]. oXiris and other adsorptive membranes combine filtration with adsorptive properties and have been reported in pediatric CAR-T-related immune-effector toxicity [48,49]. Hemoadsorption cartridges offer a technically flexible method that can be integrated into existing extracorporeal circuits or used as hemoperfusion, but comparative superiority over CKRT, plasma exchange, or adsorptive membranes has not been demonstrated. The most appropriate future trials may therefore compare protocolized standard care with or without hemoadsorption within specific syndromes, rather than comparing all extracorporeal modalities across heterogeneous diseases.

Although the evidence does not support formal recommendations, several practical lessons can be drawn. First, hemoadsorption in pediatric oncology has mainly been used as rescue adjunctive support in severe inflammatory or toxic syndromes rather than as a standardized early intervention. Second, reported biomarker reductions should be interpreted cautiously and should not be used alone to define clinical success; patient-centered outcomes, organ support, and safety must be reported alongside biomarkers. Third, syndrome-specific intent matters: cytokine burden is the target in sepsis or HLH-like hyperinflammation, drug removal is the target in delayed methotrexate clearance, and bilirubin or albumin-bound toxin removal is the target in extracorporeal liver support. Fourth, because hemoadsorption is nonselective, therapeutic drug monitoring and multidisciplinary coordination are particularly important in children receiving antimicrobials, antifungals, immunomodulators, or chemotherapy. Finally, device choice should be framed as a technical decision under uncertainty rather than as evidence-based superiority.

This review has several limitations. The evidence base is small, heterogeneous, and dominated by case reports, case series, and retrospective observational studies. The search was restricted to English-language publications in PubMed, Scopus, and Web of Science, which created potential biases related to language and database selection. Six reports identified during the screening process could not be retrieved, which may have affected the completeness of the results. Although the search strategy was expanded to include albumin dialysis and extracorporeal liver support modalities, the main synthesis remained focused on cartridge-based hemoperfusion. Therefore, this review does not comprehensively evaluate all extracorporeal detoxification approaches. Inclusion of supportive and contextual evidence required judgment. However, we attempted to reduce ambiguity by defining evidence categories a priori. The risk-of-bias assessment was limited by the poor reporting of the included studies, several of which originated from overlapping centers or author groups, creating possible patient overlap. Mortality outcomes were reported with inconsistent timeframes and could not be pooled. Finally, because many reports involved rescue therapy during complex critical illness, we could not separate the independent effect of hemoperfusion from the effects of antimicrobials, source control, immunomodulation, CRRT or CKRT, transfusion, ventilation, vasoactive support, or natural recovery.

Despite these limitations, the review identifies a clear research agenda. First, multicenter prospective registries should capture all hemoadsorption episodes in pediatric oncology, including unsuccessful treatments, to reduce publication bias and define real-world safety. Second, future interventional studies should use syndrome-specific eligibility criteria and standardized triggers for initiation. Third, core outcomes should include vasoactive-inotrope score, lactate, oxygenation, pSOFA or PELOD-2, renal recovery, cytokine and ferritin kinetics, antimicrobial and chemotherapy concentrations, bleeding and thrombosis, circuit complications, PICU-free days, hospital survival, and longer-term functional status. Fourth, pharmacokinetic substudies are essential because drug adsorption may be the most clinically important safety issue in this population [34,35]. Until such data are available, hemoadsorption should be described as individualized adjunctive rescue therapy in highly selected critically ill pediatric oncology patients, not as standard care or proven disease-modifying treatment.

## 5. Conclusions

In critically ill pediatric oncology and hemato-oncology patients, hemoadsorption has been reported as an individualized adjunctive rescue intervention for severe inflammatory or toxic syndromes, particularly sepsis, septic shock, HLH-like hyperinflammation, CAR-T-cell toxicity, delayed methotrexate clearance, and transplant-related complications. Published reports describe short-term before–after reductions in inflammatory biomarkers and selected physiologic variables, but these observations cannot be separated from concurrent intensive care interventions and do not establish treatment effect or survival benefit. Evidence remains insufficient to determine optimal timing, patient selection, device superiority, anticoagulation strategy, pharmacokinetic safety, or patient-centered benefit. Future multicenter studies should use syndrome-specific eligibility criteria, standardized timing and outcome definitions, systematic adverse-event reporting, patient-overlap checks, and therapeutic drug monitoring.

## Figures and Tables

**Table 1:** Characteristics of included studies and key reported findings.

Study	Evidence Category	Design/Population	Oncology or Hemato-Oncology Context	Indication	Device/Circuit	Key Reported Finding	Overlap Note
Milella 2019 [26]	Direct	Retrospective case series; pediatric hyperinflammation; oncology case extracted	1 oncology HLH case, age 4 y	Sepsis and multiorgan failure	CytoSorb + CVVHDF; heparin	IL-6/IL-10/PCT/CRP monitored; survived to hospital discharge	Not assessable from submitted manuscript
Bottari 2022 [25]	Direct/supportive	Case series; 6 HLH children; 2 oncologic	Burkitt lymphoma and malignancy-associated HLH subset	Sepsis-triggered HLH/MODS	CytoSorb; heparin; citrate considerations	Ferritin, IL-10, and PCT decreased; mixed mortality by HLH subtype	Unclear; mixed HLH cohort
Milella & Ficarella 2017 [27]	Supportive	Case report	Secondary HLH; malignant diagnosis not clearly specified in submitted manuscript	Septic shock and MODS	CytoSorb + CVVHDF with plasmapheresis; heparin	Survived; discharged to oncology ward	Unclear
Bottari 2020a [28]	Supportive	Retrospective observational study; pediatric septic shock	3/8 with hematologic malignancy; outcomes not separately extractable in submitted manuscript	Refractory septic shock	CytoSorb + CRRT; heparin	Overall cohort 75% survival; improved vasoactive-inotropic score	Possible institutional overlap with other Bottari reports cannot be assessed from submitted manuscript
Saeed 2025 [24]	Direct	Case report	AML secondary to Fanconi anemia	Septic shock, hyper-IL-6-naemia, thrombocytopenia	CytoSorb + CRRT; citrate	IL-6/PCT/CRP decreased; stabilized and transferred to chemotherapy ward	No overlap apparent from submitted manuscript
Ryazanova 2024 [22]	Direct	Retrospective comparative observational study; 20 oncology patients	ALL, AML, osteosarcoma, medulloblastoma, sarcoma; pure red cell aplasia excluded from malignant subset	Sepsis, cytokine storm, MODS	CytoSorb or HA330 + CVVHDF; heparin	CRP/PCT/IL-6 decreased; oxygenation and pSOFA improved; no statistically significant device difference	Possible in-stitutional overlap; cannot be assessed from submitted manuscript
Bottari 2023 [29]	Supportive	Single-arm pilot interventional study with historical controls	Mixed PICU; 52% hemato-oncologic/immune deficiency	Septic shock	CytoSorb + CKRT; citrate/heparin	Hemodynamic variables improved in overall cohort after early use	Possible institutional overlap with Bottari 2020a [25] cannot be assessed from submitted manuscript
Sazonov 2021a [23]	Direct	Case series; 3 children	Pure red cell aplasia; relapsed ALL; ALL. Two malignant leukemia cases used as direct oncology evidence	Neutropenic septic shock	HA330 + CVVHDF; heparin	CRP/PCT/IL-6 decreased; two improved/discharged; one death reported later in clinical course	Possible in-stitutional overlap; cannot be assessed from submitted manuscript
Sazonov 2021b [30]	Direct	Case report	ALL	Delayed methotrexate clearance with hepato-nephrotoxicity	HA230 + CVVHDF; heparin	Methotrexate decreased by 85.27%; discharged	Possible overlap with institutional reports unlikely
Bottari 2020b [31]	Direct	Case report	B-cell precursor ALL after CAR-T	Severe CRS and ARDS	CytoSorb + CRRT	Cytokines decreased; CRS/ARDS resolved; discharged	Possible institutional overlap with Bottari reports not assessable from submitted manuscript
Hui 2023 [32]	Supportive	Retrospective pediatric case series	Bone marrow transplant recipients	Severe hyperbilirubinemia/liver dysfunction	CytoSorb alone or with SPAD; often no anticoagulation	Median bilirubin removal 44.6%; no complications reported	No overlap apparent from submitted manuscript
Pineres-Olave 2025 [33]	Contextual	Retrospective cohort; mixed neonatal/pediatric critical illness	3/11 oncology comorbidity	Inflammation, MODS, hyperbilirubinemia, poisonings	CytoSorb or oXiris with CRRT/ECMO; citrate	VIS and lactate decreased in overall cohort; high baseline mortality risk	Contextual mixed cohort; no direct oncology inference

Note: ALL, acute lymphoblastic leukemia; AML, acute myeloid leukemia; ARDS, acute respiratory distress syndrome; CAR-T, chimeric antigen receptor T-cell; CKRT, continuous kidney replacement therapy; CRP, C-reactive protein; CRRT, continuous renal replacement therapy; CRS, cytokine release syndrome; CVVHDF, continuous veno-venous hemodiafiltration; ECMO, extracorporeal membrane oxygenation; HA230 and HA330, Jafron hemoadsorption cartridges; HLH, hemophagocytic lymphohistiocytosis; IL, interleukin; MODS, multiorgan dysfunction syndrome; PCT, procalcitonin; PICU, pediatric intensive care unit; pSOFA, pediatric Sequential Organ Failure Assessment; SPAD, single-pass albumin dialysis; VIS, vasoactive-inotropic score; y, years. Evidence categories were assigned according to extractability and relevance to pediatric oncology. “Key reported finding” summarizes temporal findings reported by the original authors and should not be interpreted as proof of treatment effect.

**Table 2:** Quantitative biomarker and physiologic response in direct pediatric oncology/HLH evidence.

Study/Subgroup	Population	Inflammatory Biomarker Response	Physiologic Response	Interpretation
Ryazanova 2024 [22], HA330	10 oncology patients	CRP 288.2 to 105.6 mg/L; PCT 245.1 to 11.9 ng/mL; IL-6 539.9 to 107.7 pg/mL	SpO_2_/FiO_2_ 204.6 to 406.7; norepinephrine 0.83 to 0.10 mcg/kg/min; pSOFA 16.6 to 3.5	Reported before–after biomarker and physiologic changes; no statistically significant device difference
Ryazanova 2024 [22], CytoSorb	10 oncology patients	CRP 242.6 to 84.6 mg/L; PCT 36.3 to 10.3 ng/mL; IL-6 322.7 to 111.5 pg/mL	SpO_2_/FiO_2_ 219.3 to 411.6; norepinephrine 0.95 to 0.10 mcg/kg/min; pSOFA 16.7 to 3.4	Reported before–after changes similar in direction to HA330 group
Sazonov 2021a [23], ALL case 1	Relapsed ALL, septic shock, AKI	CRP −65.3%; PCT −91.1%; IL-6 −47.7%	SpO_2_/FiO_2_ 158 to 466; dopamine discontinued	Reported post-session improvement in shock-related variables
Sazonov 2021a [23], ALL case 2	ALL after high-dose methotrexate, sepsis, AKI	CRP −49.9%; PCT −94.2%; IL-6 −65.5%	Oxygenation index 7.8 to 4.2; norepinephrine stopped; dopamine reduced	Initial response despite later death from refractory malignancy
Saeed 2025 [24]	AML/Fanconi anemia, invasive fungal disease	IL-6 fell within 24 h; PCT halved by 8 h; CRP declined over 40 h	Clinical stabilization and regression of skin lesions	Reported use despite thrombocytopenia; systematic safety inference not possible
Bottari 2022 [25]	6 HLH children; 2 oncologic	IL-10 140.7 to 31.6 pg/mL; PCT 56.3 to 1.93 ng/mL; ferritin 59,280 to 1578 ng/mL	VIS and PELOD numerically improved	Consistent with cytokine-reduction hypothesis; oncologic subset small
Milella 2019 [26], oncology HLH case	HLH patient	Lower baseline cytokine burden in survivor profile	8 days catecholamines; 4 days ventilation; 5 days CRRT	Survival after early initiation

Note: AKI, acute kidney injury; ALL, acute lymphoblastic leukemia; AML, acute myeloid leukemia; CRP, C-reactive protein; CRRT, continuous renal replacement therapy; HA330, Jafron hemoadsorption cartridge; HLH, hemophagocytic lymphohistiocytosis; IL, interleukin; PCT, procalcitonin; PELOD, Pediatric Logistic Organ Dysfunction; pSOFA, pediatric Sequential Organ Failure Assessment; SpO_2_/FiO_2_, peripheral oxygen saturation/fraction of inspired oxygen ratio; VIS, vasoactive-inotropic score. Biomarker and physiologic values are reported as presented in the original studies. Pre–post changes are descriptive and were not pooled because of heterogeneity in indications, timing, devices, and outcome reporting.

**Table 3:** Reported survival and mortality outcomes by report.

Report	Extractable Oncology Sample	Outcome Horizon	Reported Survival/Mortality	Interpretation
Ryazanova 2024 [22]	19 malignant oncology cases after excluding pure red cell aplasia from malignant subset	Cohort follow-up; not a uniform 28-day/PICU endpoint in submitted manuscript	4 deaths reported in the oncology cohort	Do not pool; deaths may not represent a uniform acute hemoadsorption endpoint
Sazonov 2021a [23]	2 malignant leukemia cases from 3-case series	PICU/hospital course; one later death from refractory malignancy described in submitted manuscript	1 death among 2 malignant leukemia cases; one survivor/improved/discharged	Do not pool; later malignancy-related death is not equivalent to acute PICU/28-day mortality
Saeed 2025 [24]	1 AML/Fanconi anemia case	Survival to ward transfer	Survived to transfer to chemotherapy ward	Selected single rescue case; no prognosis/effect inference
Bottari 2022 [25]	2 oncologic cases within 6 HLH children	PICU/28-day/hospital mortality as reported in original article; details require source verification	Mixed mortality by HLH subtype in submitted manuscript	Outcome should be reported by horizon after source verification
Milella 2019 [26]	1 oncology HLH case extracted	Survival to ward/hospital discharge	Survived to hospital discharge	Selected case; no effect inference
Milella & Ficarella 2017 [27]	1 secondary HLH case; malignancy not clearly specified in submitted manuscript	Survival/discharge to oncology ward	Survived; discharged to oncology ward	Supportive only unless malignancy confirmed
Bottari 2020a [28]	3/8 hematologic malignancy in mixed septic shock cohort; oncology outcomes not separately reported in submitted manuscript	Overall cohort outcome	75% survival in overall cohort	Do not interpret as pediatric oncology survival
Bottari 2023 [29]	Mixed PICU; 52% hemato-oncologic/immune deficiency	Historical-control pilot outcome reporting	Hemodynamic improvement in overall cohort; survival outcome not oncology-specific in submitted manuscript	Do not interpret as direct oncology survival
Sazonov 2021b [30]	1 ALL case	Discharge outcome	Discharged after methotrexate reduction	Toxicity indication; not comparable with sepsis/HLH mortality
Bottari 2020b [31]	1 B-cell precursor ALL CAR-T case	Discharge outcome	CRS/ARDS resolved; discharged	CAR-T toxicity indication; not comparable with sepsis/HLH mortality
Hui 2023 [32]	Bone marrow transplant recipients	Hyperbilirubinemia/liver dysfunction outcomes	Median bilirubin removal 44.6%; no complications reported	Transplant/liver-support context; not comparable with sepsis mortality
Pineres-Olave 2025 [33]	3/11 oncology comorbidity in mixed cohort	Mixed critical illness cohort outcomes	VIS and lactate decreased in overall cohort; high baseline mortality risk	Contextual only; not direct oncology survival

Note: Outcomes are summarized as reported by each publication and were not pooled because outcome horizons differed across reports and included survival to ward transfer, PICU/hospital course, discharge, and later death from malignancy progression. Reports from overlapping author groups or institutions were linked narratively when patient overlap could not be excluded. These data should not be interpreted as evidence of treatment efficacy, prognosis, or survival benefit.

## Data Availability

All extracted data used in this review are available in the manuscript tables and supplementary tables. No unpublished patient-level data were used.

## Supplementary Materials

The following supporting information can be downloaded at https://www.mdpi.com/article/10.3390/children13070961/s1, Supplementary S1: Complete database search strategies; Table S1: Methodological quality and risk-of-bias assessment of included clinical reports; Table S2: PRISMA 2020 checklist; Table S3: Detailed characteristics and overlap assessment; Table S4: Safety and adverse-event reporting.

## Abbreviations

The following abbreviations are used in this manuscript:

AKI	Acute kidney injury
ALL	Acute lymphoblastic leukemia
AML	Acute myeloid leukemia
ARDS	Acute respiratory distress syndrome
CAR-T	Chimeric antigen receptor T-cell
CI	Confidence interval
CKRT	Continuous kidney replacement therapy
CRP	C-reactive protein
CRRT	Continuous renal replacement therapy
CRS	Cytokine release syndrome
CVVHDF	Continuous veno-venous hemodiafiltration
ECMO	Extracorporeal membrane oxygenation
GRADE	Grading of Recommendations Assessment, Development and Evaluation
HA230	Jafron HA230 hemoadsorption cartridge
HA330	Jafron HA330 hemoadsorption cartridge
HA380	Jafron HA380 hemoadsorption cartridge
HDMTX	High-dose methotrexate
HLH	Hemophagocytic lymphohistiocytosis
HSCT	Hematopoietic stem-cell transplantation
ICANS	Immune effector cell-associated neurotoxicity syndrome
IL	Interleukin
IL-6	Interleukin-6
IL-8	Interleukin-8
IL-10	Interleukin-10
JBI	Joanna Briggs Institute
MARS	Molecular Adsorbent Recirculating System
MeSH	Medical Subject Headings
MODS	Multiorgan dysfunction syndrome
NOS	Newcastle-Ottawa Scale
PCT	Procalcitonin
PELOD	Pediatric Logistic Organ Dysfunction
PELOD-2	Pediatric Logistic Organ Dysfunction-2
PICOS	Population, intervention, comparator, outcomes, and study design
PICU	Pediatric intensive care unit
PRISMA	Preferred Reporting Items for Systematic Reviews and Meta-Analyses
PRISMA-S	Preferred Reporting Items for Systematic Reviews and Meta-Analyses literature search extension
PROSPERO	International Prospective Register of Systematic Reviews
pSOFA	Pediatric Sequential Organ Failure Assessment
ROBINS-I	Risk Of Bias In Non-randomized Studies-of Interventions
SPAD	Single-pass albumin dialysis
SpO_2_/FiO_2_	Peripheral oxygen saturation/fraction of inspired oxygen ratio
SWiM	Synthesis Without Meta-analysis
TDM	Therapeutic drug monitoring
TNF-α	Tumor necrosis factor-alpha
VIS	Vasoactive-inotropic score
