# Supplementary material for: Hemoadsorptionin Critically Ill Pediatric Oncology and Hemato-Oncology Patients: A Systematic Review with Structured Narrative Synthesis
Source: Children (Basel). 2026 Jul 21;13(7):961. doi: 10.3390/children13070961 (PMC13406576; doi:10.3390/children13070961)
Supplement: Supplementary file 1 [file children-13-00961-s001.zip › children-4413923-supplementary.pdf]

# Supplementary Materials

## Supplementary S1

*S1. Search strategy*

Search date: 1 June 2026. Databases: PubMed, Scopus, and Web of Science. Limits: English-language reports; 1 January 2017, through 1 June 2026. Additional sources included reference lists of eligible studies and relevant review articles. The search was expanded during revision to include albumin dialysis and extracorporeal liver-support terms, including MARS, Prometheus, SPAD, single-pass albumin dialysis, and fractionated plasma separation and adsorption. These terms were used to identify contextual extracorporeal blood purification literature; studies focused exclusively on non-cartridge modalities were not included in the direct cartridge-based hemoadsorption synthesis unless cartridge-based hemoadsorption was also used.

Core concept 1 (intervention): hemoadsorption OR haemoadsorption OR hemoperfusion OR haemoperfusion OR extracorporeal blood purification OR cytokine adsorption OR CytoSorb OR Jafron OR HA230 OR HA330 OR HA380 OR oXiris.

Core concept 2 (population): child OR children OR pediatric OR paediatric OR adolescent OR infant OR neonate.

Core concept 3 (oncology/clinical context): oncology OR cancer OR malignancy OR leukemia OR leukaemia OR lymphoma OR osteosarcoma OR medulloblastoma OR sarcoma OR bone marrow transplantation OR hematopoietic stem cell transplantation OR CAR-T OR chimeric antigen receptor OR cytokine release syndrome OR sepsis OR septic shock OR hyperinflammation OR hemophagocytic lymphohistiocytosis OR HLH OR methotrexate OR hyperbilirubinemia.

PubMed strategy: 1. hemoadsorption[tiab] OR haemoadsorption[tiab] OR hemoperfusion[tiab] OR haemoperfusion[tiab] OR "extracorporeal blood purification"[tiab] OR "cytokine adsorption"[tiab] OR CytoSorb[tiab] OR Jafron[tiab] OR HA230[tiab] OR HA-230[tiab] OR HA330[tiab] OR HA-330[tiab] OR HA380[tiab] OR HA-380[tiab] OR oXiris[tiab] OR "albumin dialysis"[tiab] OR "single-pass albumin dialysis"[tiab] OR SPAD[tiab] OR MARS[tiab] OR "molecular adsorbent recirculating system"[tiab] OR Prometheus[tiab] OR "fractionated plasma separation and adsorption"[tiab]

2. child*[tiab] OR pediatric*[tiab] OR paediatric*[tiab] OR adolescent*[tiab] OR infant*[tiab] OR neonat*[tiab]

3. oncolog*[tiab] OR cancer[tiab] OR malignan*[tiab] OR leukem*[tiab] OR leukaem*[tiab] OR lymphoma[tiab] OR osteosarcoma[tiab] OR medulloblastoma[tiab] OR sarcoma[tiab] OR "bone marrow transplant*"[tiab] OR "hematopoietic stem cell transplant*"[tiab] OR "haematopoietic stem cell transplant*"[tiab] OR CAR-T[tiab] OR "chimeric antigen receptor"[tiab] OR "cytokine release syndrome"[tiab] OR sepsis[tiab] OR "septic shock"[tiab] OR hyperinflammation[tiab] OR HLH[tiab] OR "hemophagocytic lymphohistiocytosis"[tiab] OR "haemophagocytic lymphohistiocytosis"[tiab] OR methotrexate[tiab] OR thioguanine[tiab] OR tioguanine[tiab] OR acetaminophen[tiab] OR paracetamol[tiab] OR hyperbilirubinemia[tiab] OR

hyperbilirubinaemia[tiab] OR "sinusoidal obstruction syndrome"[tiab] OR "veno-occlusive disease"[tiab] OR "acute liver failure"[tiab] OR hepatotoxicity[tiab]

4. 1 AND 2 AND 3

5. Limits: English-language reports; January 1, 2017, through June 1, 2026.

Scopus strategy

TITLE-ABS-KEY(hemoadsorption OR haemoadsorption OR hemoperfusion OR haemoperfusion OR "extracorporeal blood purification" OR "cytokine adsorption" OR CytoSorb OR Jafron OR HA230 OR "HA-230" OR HA330 OR "HA-330" OR HA380 OR "HA-380" OR oXiris OR "albumin dialysis" OR "single-pass albumin dialysis" OR SPAD OR MARS OR "molecular adsorbent recirculating system" OR Prometheus OR "fractionated plasma separation and adsorption") AND TITLE-ABS-KEY(child* OR pediatric* OR paediatric* OR adolescent* OR infant* OR neonat*) AND TITLE-ABS-KEY(oncolog* OR cancer OR malignan* OR leukem* OR leukaem* OR lymphoma OR osteosarcoma OR medulloblastoma OR sarcoma OR "bone marrow transplant*" OR "hematopoietic stem cell transplant*" OR "haematopoietic stem cell transplant*" OR "CAR-T" OR "chimeric antigen receptor" OR "cytokine release syndrome" OR sepsis OR "septic shock" OR hyperinflammation OR HLH OR "hemophagocytic lymphohistiocytosis" OR "haemophagocytic lymphohistiocytosis" OR methotrexate OR thioguanine OR tioguanine OR acetaminophen OR paracetamol OR hyperbilirubinemia OR hyperbilirubinaemia OR "sinusoidal obstruction syndrome" OR "veno-occlusive disease" OR "acute liver failure" OR hepatotoxicity)

Limits: English; 2017–2026; document types: article, review, letter, note, short survey were screened when patient-level clinical data were potentially present.

Web of Science strategy

TS=(hemoadsorption OR haemoadsorption OR hemoperfusion OR haemoperfusion OR "extracorporeal blood purification" OR "cytokine adsorption" OR CytoSorb OR Jafron OR HA230 OR "HA-230" OR HA330 OR "HA-330" OR HA380 OR "HA-380" OR oXiris OR "albumin dialysis" OR "single-pass albumin dialysis" OR SPAD OR MARS OR "molecular adsorbent recirculating system" OR Prometheus OR "fractionated plasma separation and adsorption") AND TS=(child* OR pediatric* OR paediatric* OR adolescent* OR infant* OR neonat*) AND TS=(oncolog* OR cancer OR malignan* OR leukem* OR leukaem* OR lymphoma OR osteosarcoma OR medulloblastoma OR sarcoma OR "bone marrow transplant*" OR "hematopoietic stem cell transplant*" OR "haematopoietic stem cell transplant*" OR "CAR-T" OR "chimeric antigen receptor" OR "cytokine release syndrome" OR sepsis OR "septic shock" OR hyperinflammation OR HLH OR "hemophagocytic lymphohistiocytosis" OR "haemophagocytic lymphohistiocytosis" OR methotrexate OR thioguanine OR tioguanine OR acetaminophen OR paracetamol OR hyperbilirubinemia OR hyperbilirubinaemia OR "sinusoidal obstruction syndrome" OR "veno-occlusive disease" OR "acute liver failure" OR hepatotoxicity)

Limits: English; publication date January 1, 2017, to June 1, 2026.

Records were exported to EndNote. No machine-learning automation tools were used for eligibility decisions. If any records were removed before screening for ineligibility, the rule used for removal was documented in the PRISMA flow diagram and screening log.

**Supplementary Table S1.** Methodological quality and risk-of-bias assessment of included clinical reports.

| **Study** | Tool/domains | Domain-level judgment summary | Overall confidence for causal inference | Main limitation |
|---|---|---|---|---|
| Bottari 2020b [31] | Case report; JBI case report domains | Selection: single selected case; Ascertainment: diagnosis/intervention described; Causality: very serious confounding by concurrent CAR-T toxicity management; Reporting: short follow-up | High/very serious limitations for causal inference | Single patient; multiple concurrent therapies; no comparator |
| Milella 2019 [26] | Case series; JBI case series domains | Selection: selected hyperinflammatory cases; Ascertainment: biomarkers reported; Causality: serious confounding; Reporting: oncology case extracted from heterogeneous series | High/serious limitations | Small heterogeneous series; no control group |
| Bottari 2020a [28] | Retrospective observational; NOS domains | Selection: small mixed cohort; Comparability: no untreated comparable oncology control; Outcome: overall cohort outcomes not oncology-specific | Serious limitations | Small cohort; confounding by indication; oncology outcomes not separately extractable in submitted manuscript |
| Bottari 2022 [25] | Case series; JBI case series domains | Selection: mixed HLH etiologies; Ascertainment: biomarkers reported; Causality: co-interventions and disease heterogeneity; Reporting: oncologic subset small | Serious limitations | Mixed HLH etiologies; limited power; small oncologic subset |
| Bottari 2023 [29] | Nonrandomized pilot with historical controls; ROBINS-I domains | Confounding: serious; Selection: serious; Intervention classification: probably adequate; Missing data/outcomes: unclear; Measurement: nonblinded; Reporting: unclear | Serious/critical limitations for treatment effect | Historical controls; mixed population; no oncology-specific outcome inference |

| **Study** | Tool/domains | Domain-level judgment summary | Overall confidence for causal inference | Main limitation |
|---|---|---|---|---|
| Hui 2023 [32] | Retrospective case series; JBI domains | Selection: selected hyperbilirubinemia cases; Ascertainment: bilirubin kinetics described; Causality: co-interventions and mixed modalities; Reporting: safety reporting incomplete | Serious limitations | Mixed extracorporeal modalities and indications |
| Milella & Ficarella 2017 [27] | Case report; JBI domains | Selection: single emergency case; Ascertainment: intervention described; Causality: multiple simultaneous therapies including plasmapheresis/CVVHDF; Reporting: limited standardization | High/very serious limitations | Emergency off-label intervention; no comparator |
| Pineres-Olave 2025 [33] | Retrospective cohort; NOS domains | Selection: mixed neonatal/pediatric critical illness; Comparability: no external control; Outcome: overall cohort outcomes; Directness: contextual only | Serious limitations for oncology inference | Heterogeneous indications; mixed devices; no external control |
| Ryazanova 2024 [22] | Retrospective comparative observational; NOS/ROBINS-I domains | Selection: small single/limited-center cohort; Confounding: nonrandom device allocation; Comparability: no untreated control; Outcome: survival horizon unclear for acute mortality | Serious limitations for causal inference | No untreated comparator; possible patient overlap with Sazonov 2021a |
| Saeed 2025 [24] | Case report; JBI domains | Selection: single selected case; Ascertainment: biomarker sequence described; Causality: concurrent anti-infective/supportive therapies; Reporting: follow-up limited | High/very serious limitations | Single case; severe co-interventions |
| Sazonov 2021a [23] | Case series; JBI domains | Selection: three selected cases; Ascertainment: biomarkers reported; Causality: uncontrolled rescue use; Reporting: | High/serious limitations | Only three patients; co-interventions; possible overlap with Ryazanova 2024 |

| Study | Tool/domains | Domain-level judgment summary | Overall confidence for causal inference | Main limitation |
|---|---|---|---|---|
| | | possible overlap with later institutional cohort | | |
| Sazonov 2021b [30] | Case report; JBI domains | Selection: single toxicity case; Ascertainment: methotrexate concentration reported; Causality: concurrent supportive care; Reporting: single-patient evidence | High limitations for generalization | Single case; effect may reflect concurrent care |

*HLH, hemophagocytic lymphohistiocytosis; JBI, Joanna Briggs Institute; NOS, Newcastle-Ottawa Scale; ROBINS-I, Risk Of Bias In Non-randomized Studies- of Interventions. Methodological quality ratings reflect review-level qualitative appraisal according to study design and should be interpreted in the context of small sample sizes, uncontrolled designs, heterogeneous indications, and frequent co-interventions.

**Supplementary Table S2.** PRISMA 2020 Checklist [15].

| Section and Topic | Item # | Checklist item | Location where item is reported |
|---|---|---|---|
| **TITLE** | | | |
| Title | 1 | Identify the report as a systematic review. | Page 1 |
| **ABSTRACT** | | | |
| Abstract | 2 | See the PRISMA 2020 for Abstracts checklist. | 1 |
| **INTRODUCTION** | | | |
| Rationale | 3 | Describe the rationale for the review in the context of existing knowledge. | 2-3 |
| Objectives | 4 | Provide an explicit statement of the objective(s) or question(s) the review addresses. | 3 |
| **METHODS** | | | |
| Eligibility criteria | 5 | Specify the inclusion and exclusion criteria for the review and how studies were grouped for the syntheses. | 3-4 |
| Information sources | 6 | Specify all databases, registers, websites, organisations, reference lists and other sources searched or consulted to identify studies. Specify the date when each source was last searched or consulted. | 3 |
| Search strategy | 7 | Present the full search strategies for all databases, registers and websites, including any filters and limits used. | 3,5,15 |
| Selection process | 8 | Specify the methods used to decide whether a study met the inclusion criteria of the review, including how many reviewers screened each record and each report retrieved, whether they worked independently, and if applicable, details of automation tools used in the process. | 4 |
| Data collection process | 9 | Specify the methods used to collect data from reports, including how many reviewers collected data from each report, whether they worked independently, any processes for obtaining or confirming data from study investigators, and if applicable, details of automation tools used in the process. | 4 |
| Data items | 10a | List and define all outcomes for which data were sought. Specify whether all results that were compatible with each outcome domain in each study were sought (e.g. for all measures, time points, analyses), and if not, the methods used to decide which results to collect. | 4 |
| | 10b | List and define all other variables for which data were sought (e.g. | 4 |

| Section and Topic | Item # | Checklist item | Location where item is reported |
|---|---|---|---|
| | | participant and intervention characteristics, funding sources). Describe any assumptions made about any missing or unclear information. | |
| Study risk of bias assessment | 11 | Specify the methods used to assess risk of bias in the included studies, including details of the tool(s) used, how many reviewers assessed each study and whether they worked independently, and if applicable, details of automation tools used in the process. | 4 |
| Effect measures | 12 | Specify for each outcome the effect measure(s) (e.g. risk ratio, mean difference) used in the synthesis or presentation of results. | 4-5; 7-9 |
| Synthesis methods | 13a | Describe the processes used to decide which studies were eligible for each synthesis (e.g. tabulating the study intervention characteristics and comparing against the planned groups for each synthesis (item #5)). | 3,4 |
| | 13b | Describe any methods required to prepare the data for presentation or synthesis, such as handling of missing summary statistics, or data conversions. | 4-5 |
| | 13c | Describe any methods used to tabulate or visually display results of individual studies and syntheses. | 5-9 (tables, figures) |
| | 13d | Describe any methods used to synthesize results and provide a rationale for the choice(s). If meta-analysis was performed, describe the model(s), method(s) to identify the presence and extent of statistical heterogeneity, and software package(s) used. | 4-5 |
| | 13e | Describe any methods used to explore possible causes of heterogeneity among study results (e.g. subgroup analysis, meta-regression). | 3-10 |
| | 13f | Describe any sensitivity analyses conducted to assess robustness of the synthesized results. | 8-9 |
| Reporting bias assessment | 14 | Describe any methods used to assess risk of bias due to missing results in a synthesis (arising from reporting biases). | 4 |
| Certainty assessment | 15 | Describe any methods used to assess certainty (or confidence) in the body of evidence for an outcome. | 4 |
| **RESULTS** | | | |
| Study selection | 16a | Describe the results of the search and selection process, from the number of records identified in the search to the number of studies included in the review, ideally using a flow diagram. | 5-6 |
| | 16b | Cite studies that might appear to meet the inclusion criteria, but which were excluded, and explain why they were excluded. | 5-6 |
| Study characteristics | 17 | Cite each included study and present its characteristics. | 6-7 |
| Risk of bias in studies | 18 | Present assessments of risk of bias for each included study. | 16 |
| Results of individual studies | 19 | For all outcomes, present, for each study: (a) summary statistics for each group (where appropriate) and (b) an effect estimate and its precision (e.g. confidence/credible interval), ideally using structured tables or plots. | 7-9 |
| Results of syntheses | 20a | For each synthesis, briefly summarise the characteristics and risk of bias among contributing studies. | 7-9, 16 |
| | 20b | Present results of all statistical syntheses conducted. If meta-analysis was done, present for each the summary estimate and its precision (e.g. confidence/credible interval) and measures of statistical heterogeneity. If comparing groups, describe the direction of the effect. | 8-9 |
| | 20c | Present results of all investigations of possible causes of heterogeneity among study results. | 8-9 |
| | 20d | Present results of all sensitivity analyses conducted to assess the | 8-9 |

| Section and Topic | Item # | Checklist item | Location where item is reported |
|---|---|---|---|
| | | robustness of the synthesized results. | |
| Reporting biases | 21 | Present assessments of risk of bias due to missing results (arising from reporting biases) for each synthesis assessed. | 12 |
| Certainty of evidence | 22 | Present assessments of certainty (or confidence) in the body of evidence for each outcome assessed. | 4,12,16 |
| **DISCUSSION** | | | |
| Discussion | 23a | Provide a general interpretation of the results in the context of other evidence. | 10-11 |
| | 23b | Discuss any limitations of the evidence included in the review. | 12 |
| | 23c | Discuss any limitations of the review processes used. | 12 |
| | 23d | Discuss implications of the results for practice, policy, and future research. | 12-13 |
| **OTHER INFORMATION** | | | |
| Registration and protocol | 24a | Provide registration information for the review, including register name and registration number, or state that the review was not registered. | 3 |
| | 24b | Indicate where the review protocol can be accessed, or state that a protocol was not prepared. | 3 |
| | 24c | Describe and explain any amendments to information provided at registration or in the protocol. | 3 |
| Support | 25 | Describe sources of financial or non-financial support for the review, and the role of the funders or sponsors in the review. | 13 |
| Competing interests | 26 | Declare any competing interests of review authors. | 13 |
| Availability of data, code and other materials | 27 | Report which of the following are publicly available and where they can be found: template data collection forms; data extracted from included studies; data used for all analyses; analytic code; any other materials used in the review. | 13 |

**Supplementary Table S3: detailed characteristics and overlap assessment**

| **Report** | **Evidence category** | **Country/centre** | **Recruitment dates** | **Design/sample** | **Oncology context** | **Age/sex/weight** | **Indication/severity** | **Device/circuit** | **Timing/duration** | **Anticoagulation/priming** | **Co-interventions/adverse-event ascertainment** | **Follow-up/funding/conflicts** | **Overlap assessment** |
|---|---|---|---|---|---|---|---|---|---|---|---|---|---|
| Milella 2019 [26] | Direct | Not extractable from source article | Not extractable from source article | Retrospective case series; pediatric hyperinflammation; oncology case extracted | 1 oncology HLH case | Not extractable from source article | Sepsis and multiorgan failure | CytoSorb + CVVHDF; heparin | Not extractable from source article | Not extractable from source article | Not systematically reported in submitted manuscript | Not extractable from source article | Not assessable from submitted manuscript |
| Bottari 2022 [25] | Direct/supportive | Not extractable from source article | Not extractable from source article | Case series; 6 HLH children; 2 oncologic | 2 oncologic cases within 6 HLH children | Not extractable from source article | Sepsis-triggered HLH/MODS | CytoSorb; heparin; citrate considerations | Not extractable from source article | Not extractable from source article | Not systematically reported in submitted manuscript | Not extractable from source article | Unclear; mixed HLH cohort |
| Milella & Ficarella 2017 [27] | Supportive | Not extractable from source article | Not extractable from source article | Case report | Secondary HLH; malignant diagnosis not clearly specified in submitted | Not extractable from source article | Septic shock and MODS | CytoSorb + CVVHDF with plasmapheresis; heparin | Not extractable from source article | Not extractable from source article | Not systematically reported in submitted manuscript | Not extractable from source article | Unclear |

| Report | Evidence category | Country/centre | Recruitment dates | Design/sample | Oncology context | Age/sex/weight | Indication/severity | Device/circuit | Timing/duration | Anticoagulation/priming | Co-interventions/adverse-event ascertainment | Follow-up/funding/conflicts | Overlap assessment |
|---|---|---|---|---|---|---|---|---|---|---|---|---|---|
| | | | | | manuscript | | | | | | | | |
| Bottari 2020a [28] | Supportive | Not extractable from source article | Not extractable from source article | Retrospective observational study; pediatric septic shock | 3/8 with hematologic malignancy; outcomes not separately extractable in submitted manuscript | Not extractable from source article | Refractory septic shock | CytoSorb + CRRT; heparin | Not extractable from source article | Not extractable from source article | Not systematically reported in submitted manuscript | Not extractable from source article | Possible institutional overlap with other Bottari reports cannot be assessed from submitted manuscript |
| Saeed 2025 [24] | Direct | Not extractable from source article | Not extractable from source article | Case report | AML secondary to Fanconi anemia | Not extractable from source article | Septic shock, hyper-IL-6-naemia, thrombocytopenia | CytoSorb + CRRT; citrate | Not extractable from source article | Not extractable from source article | Not systematically reported in submitted manuscript | Not extractable from source article | No overlap apparent from submitted |

| Report | Evidence category | Country/centre | Recruitment dates | Design/sample | Oncology context | Age/sex/weight | Indication/severity | Device/circuit | Timing/duration | Anticoagulation/priming | Co-interventions/adverse-event ascertainment | Follow-up/funding/conflicts | Overlap assessment |
|---|---|---|---|---|---|---|---|---|---|---|---|---|---|
| | | | | | | | | | | | | | manuscript |
| Ryazanova 2024 [22] | Direct | Not extractable from source article | Not extractable from source article | Retrospective comparative observational study; 20 oncology patients | ALL, AML, osteosarcoma, medulloblastoma, sarcoma; pure red cell aplasia excluded from malignant subset | Not extractable from source article | Sepsis, cytokine storm, MODS | CytoSorb or HA330 + CVVHDF; heparin | Not extractable from source article | Not extractable from source article | Not systematically reported in submitted manuscript | Not extractable from source article | Possible overlap with Sazonov 2021a [20] should be assessed |
| Bottari 2023 [29] | Supportive | Not extractable from source article | Not extractable from source article | Single-arm pilot interventional study with historical controls | Mixed PICU; 52% hemato-oncologic/immune deficiency | Not extractable from source article | Septic shock | CytoSorb + CRRT; citrate/heparin | Not extractable from source article | Not extractable from source article | Not systematically reported in submitted manuscript | Not extractable from source article | Possible institutional overlap with Bottari 2020a [25] cannot be assessed |

| Report | Evidence category | Country/centre | Recruitment dates | Design/sample | Oncology context | Age/sex/weight | Indication/severity | Device/circuit | Timing/duration | Anticoagulation/priming | Co-interventions/adverse-event ascertainment | Follow-up/funding/conflicts | Overlap assessment |
|---|---|---|---|---|---|---|---|---|---|---|---|---|---|
| | | | | | | | | | | | | | from submitted manuscript |
| Sazonov 2021a [23] | Direct | Not extractable from source article | Not extractable from source article | Case series; 3 children | Pure red cell aplasia; relapsed ALL; ALL. Two malignant leukemia cases used as direct oncology evidence | Not extractable from source article | Neutropenic septic shock | HA330 + CVVHDF; heparin | Not extractable from source article | Not extractable from source article | Not systematically reported in submitted manuscript | Not extractable from source article | Possible overlap with Ryazanova 2024 [19] should be assessed |
| Sazonov 2021b [30] | Direct | Not extractable from source article | Not extractable from source article | Case report | ALL | Not extractable from source article | Delayed methotrexate clearance with hepato-nephrotoxicity | HA230 + CVVHDF; heparin | Not extractable from source article | Not extractable from source article | Not systematically reported in submitted manuscript | Not extractable from source article | Possible overlap with institutional reports unlikely but |

| Report | Evidence category | Country/centre | Recruitment dates | Design/sample | Oncology context | Age/sex/weight | Indication/severity | Device/circuit | Timing/duration | Anticoagulation/priming | Co-interventions/adverse-event ascertainment | Follow-up/funding/conflicts | Overlap assessment |
|---|---|---|---|---|---|---|---|---|---|---|---|---|---|
| | | | | | | | | | | | | | not assessable from submitted manuscript |
| Bottari 2020b [31] | Direct | Not extractable from source article | Not extractable from source article | Case report | B-cell precursor ALL after CAR-T | Not extractable from source article | Severe CRS and ARDS | CytoSorb + CRRT | Not extractable from source article | Not extractable from source article | Not systematically reported in submitted manuscript | Not extractable from source article | Possible institutional overlap with Bottari reports not assessable from submitted manuscript |
| Hui 2023 [32] | Supportive | Not extractable from | Not extractable from | Retrospective pediatric case series | Bone marrow transplan | Not extractable from | Severe hyperbilirubinemia/liver dysfunction | CytoSorb alone or with SPAD; often no | Not extractable from | Not extractable from source article | Not systematically reported in | Not extractable from | No overlap apparent |

| **Report** | **Evidence category** | **Country/centre** | **Recruitment dates** | **Design/sample** | **Oncology context** | **Age/sex/weight** | **Indication/severity** | **Device/circuit** | **Timing/duration** | **Anticoagulation/priming** | **Co-interventions/adverse-event ascertainment** | **Follow-up/funding/conflicts** | **Overlap assessment** |
|---|---|---|---|---|---|---|---|---|---|---|---|---|---|
| | | source article | source article | | t recipients | source article | | anticoagulation | source article | | submitted manuscript | source article | from submitted manuscript |
| Pineres-Olave 2025 [33] | Contextual | Not extractable from source article | Not extractable from source article | Retrospective cohort; mixed neonatal/pediatric critical illness | 3/11 oncology comorbidity | Not extractable from source article | Inflammation, MODS, hyperbilirubinemia, poisonings | CytoSorb or oXiris with CRRT/ECMO; citrate | Not extractable from source article | Not extractable from source article | Not systematically reported in submitted manuscript | Not extractable from source article | Contextual mixed cohort; no direct oncology inference |

*" Not extractable.." indicates that the variable was not extractable from the source publication after review of the available full text. It does not indicate that the information was omitted only from the present manuscript. Where possible patient overlap was suspected, reports were linked narratively rather than combined into a pooled patient denominator.

**Supplementary Table S4: safety and adverse-event reporting**

| Report | Anticoagulation | Bleeding/thrombocytopenia | Circuit clotting/interruption | Hemodynamic intolerance | Catheter/transfusion issues | Cartridge failure | Drug/biomarker monitoring | Safety interpretation |
|---|---|---|---|---|---|---|---|---|
| Milella 2019 [26] | Heparin | Not systematically reported in submitted manuscript | Not systematically reported | Not systematically reported | Not systematically reported | Not systematically reported | IL-6/IL-10/PCT/CRP monitored; drug levels not reported | Safety cannot be determined |
| Bottari 2022 [25] | Heparin; citrate considerations | Not systematically reported | Not systematically reported | Not systematically reported | Not systematically reported | Not systematically reported | IL-10/PCT/ferritin reported; drug levels not reported | Safety cannot be determined |
| Milella & Ficarella 2017 [27] | Heparin | Not systematically reported | Not systematically reported | Not systematically reported | Not systematically reported | Not systematically reported | Drug levels not reported | Safety cannot be determined |
| Bottari 2020a [28] | Heparin | Not systematically reported | Not systematically reported | Not systematically reported | Not systematically reported | Not systematically reported | Drug levels not reported | Safety cannot be determined from oncology subset |
| Saeed 2025 [24] | Citrate | Severe thrombocytopenia present at baseline | Not systematically reported in submitted manuscript | Not systematically reported | Not systematically reported | Not systematically reported | IL-6/PCT/CRP monitored; anti-infective drug levels not extractable from source article | Reported use despite thrombocytopenia; no general safety inference |
| Ryazanova 2024 [22] | Heparin | Not systematically reported | Not systematically reported | Not systematically reported | Not systematically reported | Not systematically reported | CRP/PCT/IL-6 reported; drug levels not reported | Safety cannot be determined; comparative adverse-event data needed |

| Report | Anticoagulation | Bleeding/thrombocytopenia | Circuit clotting/interruption | Hemodynamic intolerance | Catheter/transfusion issues | Cartridge failure | Drug/biomarker monitoring | Safety interpretation |
|---|---|---|---|---|---|---|---|---|
| Bottari 2023 [29] | Citrate/heparin | Not systematically reported | Not systematically reported | Not systematically reported | Not systematically reported | Not systematically reported | Drug levels Not extractable from source article | Safety cannot be determined in oncology subset |
| Sazonov 2021a [23] | Heparin | Not systematically reported | Not systematically reported | Not systematically reported | Not systematically reported | Not systematically reported | CRP/PCT/IL-6 reported; drug levels not reported | Safety cannot be determined |
| Sazonov 2021b [30] | Heparin | Not systematically reported | Not systematically reported | Not systematically reported | Not systematically reported | Not systematically reported | Methotrexate concentration decreased by 85.27% | Drug removal was intended therapeutic target |
| Bottari 2020b [31] | Not extractable from source article | Not systematically reported | Not systematically reported | Not systematically reported | Not systematically reported | Not systematically reported | Cytokines monitored; drug levels not reported | Safety cannot be determined |
| Hui 2023 [32] | Often no anticoagulation | No complications reported in submitted manuscript | Not systematically reported | Not systematically reported | Not systematically reported | Not systematically reported | Bilirubin removal reported | Safety signal only; active adverse-event ascertainment unclear |
| Pineres-Olave 2025 [33] | Citrate | Not systematically reported | Not systematically reported | Not systematically reported | Not systematically reported | Not systematically reported | VIS/lactate reported; drug levels not reported | Contextual safety only |

Absence of reported adverse events should not be interpreted as evidence of safety. Most included reports were not designed to actively ascertain adverse events or quantify event rates. NR/not reported means the information was not available in the submitted manuscript and should be checked against the original publication if needed.
